# Childhood Trauma in Clozapine-Resistant Schizophrenia: Prevalence, and Relationship With Symptoms

**DOI:** 10.1093/schizbullopen/sgad030

**Published:** 2023-11-13

**Authors:** Robert Dudley, Douglas Turkington, Naomi Coulthard, Melissa Pyle, Andrew Gumley, Matthias Schwannauer, David Kingdon, Anthony P Morrison

**Affiliations:** Early Intervention in Psychosis Service, Cumbria, Northumberland, Tyne and Wear NHS Foundation Trust, Gosforth, Newcastle Upon Tyne, NE3 3XT, UK; Department of Psychology, University of York, York, YO10 5DD UK; Early Intervention in Psychosis Service, Cumbria, Northumberland, Tyne and Wear NHS Foundation Trust, Gosforth, Newcastle Upon Tyne, NE3 3XT, UK; Early Intervention in Psychosis Service, Cumbria, Northumberland, Tyne and Wear NHS Foundation Trust, Gosforth, Newcastle Upon Tyne, NE3 3XT, UK; Psychosis Research Unit, Greater Manchester Mental Health NHS Foundation Trust, Prestwich, M25 3BL, UK; School of Health & Wellbeing, University of, Glasgow Clarice Pears Building, 90 Byres Road, Glasgow G12 8TB UK; Department of Clinical Psychology, School of Health in Social Science, The University of Edinburgh, Old Medical School, Teviot Place, Edinburgh, EH8 9AG, UK; University Department of Psychiatry, University of Southampton, Academic Centre, College Keep 4-12 Terminus Terrace Southampton SO14 3DT, UK; Psychosis Research Unit, Greater Manchester Mental Health NHS Foundation Trust, Prestwich, M25 3BL, UK

**Keywords:** trauma, childhood abuse, childhood neglect, psychosis

## Abstract

**Background and Hypothesis:**

The role of early adversity and trauma is increasingly recognized in psychosis but treatments for trauma and its consequences are lacking. Psychological treatments need to understand the prevalence of these experiences, the relationship with specific symptoms and identify potentially tractable processes that may be targeted in therapy. It was hypothesized that greater adversity, and specifically abuse rather than neglect, would be associated with positive symptoms and specifically hallucinations. In addition, negative beliefs would mediate the relationship with positive symptoms.

**Study Design:**

292 Patients with treatment resistant psychosis completed measures of early adversity as well as current symptoms of psychosis.

**Study Results:**

Early adversity in the form of abuse and neglect were common in one-third of the sample. Adversity was associated with higher levels of psychotic symptoms generally, and more so with positive rather than negative symptoms. Abuse rather than neglect was associated with positive but not with negative symptoms. Abuse rather than neglect was associated with hallucinations but not delusions. Abuse and neglect were related to negative beliefs about the self and negative beliefs about others. Mediation demonstrated a general relationship with adversity, negative-self, and other views and overall psychotic symptoms but not in relation to the specific experience of abuse and hallucinations. Females were more likely to be abused, but not neglected, than males.

**Conclusions:**

Whilst most relationships were modest, they supported previous work indicating that adversity contributes to people with psychosis experiencing distressing symptoms especially hallucinations. Treatments need to address and target adversity.

## Introduction

Early adversity such as childhood neglect (CN) or abuse is associated with an increased risk of a range of mental health issues^1^ including psychosis.^2^ Varese and colleagues^3^ in their meta-analysis found that early adversity was strongly associated with increased risk for psychosis and that people with schizophrenia are 2.72 times more likely to have experienced adverse childhood events than healthy individuals. Evidence supports a causal role as longitudinal studies indicate early adversity precedes the onset of psychosis.^4,5^

Importantly, the nature of the adversity affects the chance of developing psychotic symptoms, as there is a stronger link between childhood abuse (CA) and later psychosis than CN.^6^ Specifically, greater endorsement of CA experiences has been linked to higher positive symptom ratings but not with negative symptoms.^7^ It is probable that early CA and neglect contribute differently to the risk of and expression of psychosis. Neglect may increase the risk of atypical neurodevelopment^8^ and childhood physical neglect (CPN) is positively correlated with negative symptoms scores.^9^ Conversely, childhood sexual abuse (CSA) is associated with greater positive symptom reporting,^10^ particularly with auditory hallucinations,^3^ ­especially in conjunction with dissociation^11^ and paranoia.^12^ However, the relationship may be modest or small. For example, Wang and colleagues^13^ ­reported correlations of *r* = .17 between childhood trauma (CT) and hallucinations in people in first episode psychosis services. Seemingly then, trauma/adversity is an important factor for some, but it is not likely to be necessary or sufficient to explain the experience of psychosis.^14^ Current estimates suggest CT plays a role for one-third of cases.^3,15,16^

Given the role for trauma and adversity it has been proposed that there is a traumatic psychosis group, characterized by positive symptoms and high levels of anxiety who are regarded as a group resistant to or non-responsive to medications^17,18^ and existing psychological therapies.^19^ Patients with severe mental illness and a higher number of stressful childhood experiences receive higher doses of antipsychotic medication and mood stabilizers.^20^ A meta-analytic review by Thomas and colleagues^21,22^ found patients with histories of childhood maltreatment were more likely than those without such experiences to have a less favorable treatment outcome.

Severe symptoms unresponsive to treatments are core features of what is termed Treatment Resistant Schizophrenia (TRS) or Treatment Resistant Psychosis (TRP). TRS is defined as schizophrenia treated over two periods with different antipsychotics at an adequate dose for at least 4 weeks, and symptoms are not reduced by at least 20%. Clozapine treatment is a more widely applied criterion that could be used as a proxy for TRS/TRP, as typically patients offered a trial of clozapine have not responded to at least two other antipsychotics.^23^ People with TRP have more severe symptoms and lower quality of life than non-treatment resistant patients. A meta-analysis by Vargas and colleagues^24^ found a significant negative relationship between CT and both overall cognition and working memory in individuals with psychotic disorders. Wells and colleagues examined eight hundred thirty-six people with schizophrenia and healthy controls from the Australian Schizophrenia Research Bank who were assessed with the Childhood Adversity Questionnaire.^25^ Higher reported levels of childhood adversity were associated with an earlier onset and persistence of impaired cognitive functioning, which are also characteristics of TRP. Some studies suggest that TRP may be more influenced by genetic vulnerabilities^26^ but it is important to establish if rates of trauma and adversity are as common as in other groups of people with psychosis to help prevent the premature closure of exploring the contribution of these experiences to TRP.^15^

There is a higher prevalence of abuse and adversity in women with psychosis.^27^ Women with psychosis reported more sexual or physical abuse than women control participants, which was not the case for men.^28^ Obviously, the presence of these experiences can have a pronounced impact on the longer-term outcome of both women and men. However, studies considering the impact of traumatic life events on psychosis symptoms have yielded inconsistent findings.^29^ Gender has been shown to moderate the relationship between early adversity and established psychosis and subclinical psychotic experiences,^30^ whereas others reveal no impact of sex/gender.^16^

Where early adversity is part of a person’s route to psychosis it is vital to study candidate mechanisms or mediators that help explain how these adverse events led to an increased chance of reporting psychotic symptoms as these may be mechanisms that can also be targeted in therapy.^6^ Potential factors that have been considered include the impact of childhood adversity/trauma on attachment relationships,^31^ affective dysregulation,^32^ and how the person coped with the trauma (eg, dissociation^11^) at the time, and subsequently. However, a commonly studied area is to do with the schemas or beliefs the person holds of the self and others.^33,34^

Cognitive models of psychosis have long proposed that negative views of self and others are important in understanding psychotic symptoms. For instance, beliefs that others are powerful and the person is weak help explain the impact of command hallucinations.^35^ Similarly, negative beliefs that others are untrustworthy, cruel, and unkind are seen as the lens from which people with paranoia view current events and helps explain the high levels of suspiciousness and mistrust.^36^ Hardy and colleagues^37^ found that negative-other beliefs (but not negative-self beliefs) mediated the relationship between childhood emotional abuse and delusions. A systematic review and meta-analysis of this topic indicates that negative-self and other beliefs mediate the relationship between developmental trauma, especially emotional abuse and neglect, and paranoia in adulthood.^38,39^

Another review of this field argued that there was strong evidence for the contribution of trauma and beliefs about self and others to psychosis symptoms. However, this was more evident in the general population than in clinical groups.^40^ In the clinical studies, six reported evidence for a mediating role of negative-self and others beliefs but five did not indicating the need for additional consideration of this issue.

In this context, the present study aims to address the prevalence of adverse experiences in a large sample of people with TRP, and consider the nature of these experiences by examining the rates of reported childhood sexual, physical, and emotional abuse (CSA, CPA, CEA) and childhood physical and emotional neglect (CPN, CEN). Also, it examines the relationship between childhood adversity and symptoms of psychosis (positive and negative symptoms, hallucinations, and delusions) and psychological variables that may be potential mediators/targets for therapy.

Given the existing literature it is possible to specify a number of hypotheses that are replications of previous work but extended to this distinct sample of people with TRP. Hence, it was predicted that:

1 Exposure to early abuse and neglect will be associated with increased psychotic symptomatology.
2 Exposure to abuse and neglect will have a greater association with positive than negative symptoms.
3 Exposure to abuse will have a greater association with psychotic symptoms than neglect.
4 Exposure to abuse will have a greater association with positive symptoms than negative symptoms.
5 Exposure to neglect will have a greater association with negative symptoms than positive symptoms.
6 Exposure to abuse will be associated with hallucinations, and delusional ideation.
7 Exposure to abuse and neglect will be associated with greater negative beliefs about self and others.

Given past research,^32,40^ it was predicted that negative beliefs about self and others would mediate the relationship between adversity and psychotic symptoms. In light of the findings in relation to the above predictions, the nature of specific relationships between adversity and symptoms can be explored using mediation. Given the uncertainty about the impact of sex this is also examined.

## Methods

### Participants

292 (210 males, and 82 females) participants were drawn from the 487 patients recruited for the FOCUS trial^41^ which aimed to determine whether cognitive behavioral therapy (CBT) is an effective treatment for Clozapine-Resistant Schizophrenia (CRS). Participants in the present study were aged between 16 and 64 years of age (*M* 42.31 SD =10.30) and either had an ICD-10 confirmed diagnosis of schizophrenia, schizoaffective disorder, or delusional disorder (schizophrenia-spectrum disorders), or met criteria for an early intervention for psychosis service (operationally defined using the PANSS^42^). Participants needed to report persistent symptoms despite an adequate trial of clozapine in terms of dose, duration, and adherence, defined as treatment with clozapine at a stable dose of 400 mg or more (unless limited by tolerability) for at least 12 weeks, or if currently augmented with a second antipsychotic that had been given for at least 12 weeks, without remission of psychotic symptoms, or discontinuation of clozapine because of adverse reactions or inefficacy in the past 24 months. The participants were predominantly white (92%), unemployed (83%), diagnosed with Schizophrenia (87%) with 90% being prescribed Clozapine.

### Design

For these analyses single group correlational design was utilized.

## Measures

### The Childhood Trauma Questionnaire

The short-form Childhood Trauma Questionnaire (CTQ)^43^ was used to assess CT exposure. This is a retrospective self-report questionnaire containing 28 items taken from the original 70-item version^44,45^ containing 5 items to assess each of the 5 main types of childhood adversities: CEN, CPN, CEA, CPA, CSA. Three additional “minimisation and deception” items were used to detect over-idealization of childhood experiences as a potential indicator of defence/minimization (eg, “I had the perfect childhood”). Each item is rated on a 5-point Likert scale scored from 0 to 4 (never true, rarely true, sometimes true, often true) where a score of 4 would represent the highest frequency for a given item. Most items are phrased in an objective way (eg, “when I was growing up someone touched me in a sexual way or made me touch them”), but others involve more subjective evaluation (eg, “when I was growing up I believe I was sexually abused”). Studies have shown adequate reliability and validity of the CTQ in measuring and differentiating the 5 types of CT^43–45^ generally and in people with emotional disorders.^46^

### The Positive and Negative Syndrome Scale

The Positive and Negative Syndrome Scale (PANSS),^42^ was used to measure symptoms of psychosis. This is a clinician-rated 30-item semi-structured interview consisting of 7 items assessing positive symptoms (hallucinations, delusions, conceptual disorganization), 7 items assessing negative symptoms (blunted affect, passive/apathetic social avoidance), and 16 items assessing general psychopathology (anxiety, depression, lack of insight, guilt). Items are scored on a 7-point Likert scale (absent, minimal, mild, moderate, moderate-severe, severe, extreme) where a score of 7 on a given item would represent highest severity. There is good evidence for the reliability and validity of the PANSS.^42^

### Psychotic Symptom Rating Scales

The Psychotic Symptom Rating Scales (PSYRATS)^47^ was used to assess dimensions of auditory hallucinations and delusional beliefs. These are semi-structured interviews with 11 items assessing the characteristics of auditory hallucinations (frequency, duration, controllability, loudness, location, severity and intensity of distress, amount and degree of negative content, beliefs about origin of voices, and disruption) and 6 items assess the characteristics of delusions (duration and frequency of preoccupation, intensity of distress, amount of distressing content, conviction, and disruption). Previous research has supported the reliability and validity of this measure amongst individuals with chronic schizophrenia.^47^

### Brief Core Scheme Scale

The Brief Core Scheme Scale (BCSS)^48^ was used to measure beliefs about self and others. This is a self-report questionnaire, where 24 items are used to assess 4 subscales: negative-self-belief (eg, “I am unloved”), negative-other belief (eg, “other people are hostile”), positive self-belief (eg, “I am valuable”), and positive other belief (eg, “others are good”). Corresponding statements are scored on a 4-point Likert scale from 0 to 4 (do not believe it, believe it slightly, believe it moderately, believe it very much, believe it totally). Previous research has demonstrated good reliability of the BCSS.^48^

### Procedure

Written informed consent was obtained and all the measures were completed with a research assistant trained in their administration. The PANSS and other outcome measures were undertaken at baseline and at the end of 9 months (end of the intervention) which is when the CTQ was undertaken. To avoid any impact of treatment, the baseline variables were utilized in the analysis. Additional information gathered included age, sex (as reported by the person), ethnicity, and diagnosis.

### Ethics

The original study was approved by NHS ethics National Research Ethics Committee (NRES Committee Northwest-Lancaster) (12/NW/0520) and relevant NHS Trust Research and Development Departments (Greater Manchester West Mental Health NHS Foundation Trust) and the trial (ISRCTN99672552) and the protocol^49^ were registered and published in advance of completion of data collection.

### Data Preparation

Missing data was not replaced and was excluded on an analysis by analysis (pairwise basis). Outliers were retained. The skewness, kurtosis, and the Kolomogorove–Smirnov values indicated a violation of the assumption of normality for the CTQ, and efforts to transform this were not successful, but the impact of this reduces in larger samples.^50^

### Statistical Analysis

Given the aims of the research the analysis consisted of 4 main steps. First, the characteristics of the included sample are considered in comparison to those FOCUS participants who did not complete the CTQ. Then the prevalence and nature of adversity is considered in this sample with consideration of similarities to past research with other groups of people with Psychosis. With regards the relationship between adversity and psychosis (hypotheses 1–7) the data was analysed using SPSS (Version 24, IBM Corp, Armonk, New York, United States). Spearman’s correlations explored the relationships between the variables (as the distributions of the CTQ did not meet assumptions for parametric analyses). Whilst the hypotheses are pre-specified there are multiple comparisons and to avoid type II error confidence intervals (CIs) and uncorrected probabilities are reported but for hypotheses 1–6 we have also indicated where these survive a strict Bonferroni correction on the basis that we have performed 12 main tests (0.05/12= 0.004). To avoid repetition where analyses remain significant with this correction it is indicated with an asterisk. The impact of sex on these relationships was also explored.

Finally, mediation analysis explored CT, negative beliefs about self and others and Psychosis symptoms using PROCESS software (mediation model 4, version 3.5.3^51^). Also, in light of the examination of the hypotheses (1–7) the most pertinent relationships between adversity and psychosis symptoms were explored in a mediation. Given the issue with the non-normal distribution of the CTQ, bootstrapping was used and the limitations of this approach are acknowledged.^52^

## Results

As can be seen in table 1 there were no differences between those FOCUS participants who did (*n* = 292) or did not complete (*n* = 195) the CTQ in terms of age, sex, or symptoms of psychosis.

**Table 1.** Descriptive Statistics (Mean and Standard Deviation) Comparing Characteristics of Those Who Completed the CTQ or Not

Measures	Completed *M* (SD) *N* = 292	Not completed *M* (SD) *N* = 195	Test	*P*
Age	42.31 (10.30)	42.72 (10.94)	*t* (485) = 0.42	.68
Sex	Males 210 (72%)	Males 136 (71%)	χ^2^ (1, 487) = 0.2	.88
	Females 82 (28%)	Females 56 (29%)		
PANSS Total	82.50 (14.22)	83.87 (13.18)	*t* (485) = 1.07	.28
PANSS Positive	24.91 (5.89)	24.97 (5.74)	*t* (485) = 1.02	.92
PANSS Negative	19.12 (5.89)	19.76 (6.68)	*t* (485) = 1.11	.28
PSYRATS Hallucinations	22.54 (13.78)	23.55 (13.15)	*t* (412) = 0.73	.47
PSYRATS Delusions	14.48 (5.44)	14.74 (5.58)	*t* (452) = 0.49	.62

*Note*: CTQ, Childhood Trauma Questionnaire; CPA, childhood physical abuse; CSA, childhood sexual abuse; CEA, childhood emotional abuse; CPN, childhood physical neglect; CEN, childhood emotional neglect.

The rates of childhood adversity in people with TRP are reported in table 2. These are higher than those reported in non-clinical participants^46^ and comparable to those reported by other groups of people with psychiatric diagnoses^1^ and groups of people with psychosis.^53^ There is a higher reporting of abuse and overall adversity in females.

**Table 2.** Descriptive Statistics (Mean and Standard Deviation) and Mann–Whitney *U* Tests, Comparing Childhood Trauma Questionnaire Scores Between Males and Females

CTQ Measures	Males *M* (SD)	Females *M* (SD)	*U*	*P*
CTQ Total	*N* = 210	*N* = 82		
	43.86 (17.83)	52.72 (24.24)	7076.00	.018
CTQ Abuse	*N* = 215	*N* = 86		
	23.83 (11.09)	31.29 (16.47)	7099.50	.002
CTQ Neglect	*N* = 228	*N* = 86		
	19.99 (8.51)	21.53 (9.10)	8821.50	.170
CPA	*N* = 229	*N* = 90		
	7.47 (4.04)	8.96 (5.23)	9156.50	.099
CSA	*N* = 227	*N* = 89		
	7.18 (4.79)	10.44 (7.83)	8505.50	.007
CEA	*N* = 229	*N* = 90		
	9.32 (4.85)	11.99 (5.71)	7460.00	.000
CPN	*N* = 231	*N* = 90		
	8.64 (3.98)	8.87 (4.07)	10033.50	.622
CEN	*N* = 230	*N* = 88		
	11.33 (5.30)	12.56 (5.80)	8875.50	.089

*Note*: CTQ, Childhood Trauma Questionnaire; CPA, childhood physical abuse; CSA, childhood sexual abuse; CEA, childhood emotional

abuse; CPN, childhood physical neglect; CEN, childhood emotional neglect.

Whilst reporting high total scores within the sample there was variation in the rates reported of adverse experiences. As is evident in table 3, when considered by extent of adversity it is apparent that between 40% and 70% reported none to minimal experiences of neglect and abuse. These findings are broadly in line with a meta-analysis of 23 studies demonstrating that in people with psychosis the prevalence of self-reported CSA was 26%, physical abuse 39%, and emotional abuse 36%.^54^ When adversity is reported, there are often quite noticeable levels of severe to extreme levels that are generally more frequent in females than males, and noticeably so in relation to abuse.

**Table 3.** Descriptive Statistics (Number and Percentage) of Types and Levels of Abuse Reported by Males and Females Within the Childhood Trauma Questionnaire

CTQ Subscales	Males *N* (%)	Females *N* (%)	Total *N* (%)
Physical abuse	*N* = 229	*N* = 90	*N* = 319
None to minimal	158 (69%)	50 (55.6%)	208 (65.2%)
Minimal to moderate	28 (12.2%)	10 (11.1%)	38 (11.9%)
Moderate to severe	20 (8.7%)	8 (8.9%)	28 (8.8%)
Severe to extreme	23 (10%)	22 (24.4%)	45 (14.1%)
Sexual abuse	*N* = 227	*N* = 89	*N* = 316
None to minimal	166 (73.1%)	56 (62.9%)	222 (70.3%)
Minimal to moderate	12 (5.3%)	0 (0%)	12 (3.8%)
Moderate to severe	21 (9.3%)	5 (5.6%)	26 (8.2%)
Severe to extreme	28 (12.3%)	28 (31.5%)	56 (17.7%)
Emotional abuse	*N* = 229	*N* = 90	*N* = 319
None to minimal	124 (54.1%)	28 (31.1%)	152 (47.6%)
Minimal to moderate	55 (24%)	25 (27.8%)	80 (25.1%)
Moderate to severe	22 (9.6%)	11 (12.2%)	33 (10.3%)
Severe to extreme	28 (12.2%)	26 (28.9%)	54 (16.9%)
Physical neglect	*N* = 231	*N* = 90	*N* = 321
None to minimal	116 (50.2%)	40 (44.4%)	156 (48.6%)
Minimal to moderate	39 (16.9%)	23 (25.6%)	62 (19.3%)
Moderate to severe	36 (15.6%)	11 (12.2%)	47 (14.6%)
Severe to extreme	40 (17.3%)	16 (17.8%)	56 (17.4%)
Emotional neglect	*N* = 230	*N* = 88	*N* = 318
None to minimal	99 (43%)	28 (31.8%)	127 (39.9%)
Minimal to moderate	73 (31.7%)	32 (36.4%)	105 (33%)
Moderate to severe	23 (10%)	11 (12.5%)	34 (10.7%)
Severe to extreme	35 (15.2%)	17 (19.3%)	52 (16.4%)

With regards the hypotheses these are examined in turn and the data is reported in table 4. Hypothesis 1 predicted that exposure to early adversity (abuse and neglect) would be associated with increased psychotic symptomatology. This was supported (CTQ total, and PANSS total *r*(292) =.21, *P* < .001* (CIs .10 .32)). Consistent with hypothesis two, the relationship was with positive symptoms rather than negative symptoms (CTQ total, and PANSS Positive *r*(292) = .17, *P* = .004*, (CIs. 05. 28), PANSS Negative *r*(292) = .046, *P* = .435 (CIs −.07 .16)).

**Table 4.** Descriptive Statistics (Means, Standard Deviations) and Correlations Between Childhood Trauma Questionnaire and PANSS/PSYRATS Symptoms of Psychosis

Measures	*M* (SD)	1	2	3	4	5	6	7	8
		*r*, *P*, *N*	*r*, *P*, *N*	*r*, *P*, *N*	*r*, *P*, *N*	*r*, *P*, *N*	*r*, *P*, *N*	*r*, *P*, *N*	*r*, *P*, *N*
1. Total CTQ	46.34 (20.19)	1							
		292							
2. CTQ Abuse	25.96 (13.26)	.91**	1						
		.000							
		292	301						
3. CTQ Neglect	20.41 (8.69)	.89**	.64**	1					
			.000	.000					
		292	292	314					
4. PANSS Total	83.05 (13.82)	.21**	.24**	.15*	1				
			.000	.000	.009				
		292	301	314	487				
5. PANSS Positive	24.94 (5.83)	.17*	.22**	.09	.61**	1			
			.004	.000	.125	.000			
		292	301	314	487	487			
6. PANSS Negative	19.38 (6.22)	.05	.04	.06	.57**	.14*	1		
			.435	.521	.313	.000	.002		
		292	301	314	487	487	487		
7. PSYRATS Hallucinations	22.91 (13.54)	.12*	.16*	.04	.21**	.21**	.07	1	
			.048	.008	.56	.000	.000	.184	
		261	270	280	414	414	414	414	
8. PSYRATS Delusions	14.59 (5.49)	.07	.10	.02	.39**	.43**	.11*	.31**	1
			.271	.085	.794	.000	.000	.019	.000
		275	284	295	454	454	454	392	454

Note: **Indicates *P* < .001.

*Indicates *P* < .05.

Hypothesis 3 examined how exposure to abuse and neglect would relate to psychotic symptoms. Abuse has a greater relationship with psychotic symptoms (CTQ Abuse and PANSS total *r*(301) = .24, *P* < .001*, (CIs .13. 35)) than neglect (CTQ Neglect and PANSS total *r*(314) = .15, *P* = .009, (CIs .03 .26)).

Hypothesis 4, as predicted, demonstrated that abuse (CTQ Abuse and PANSS Positive *r*(301) = .22, *P* = .001*, (CIs .11 .33)), rather than neglect (CTQ Neglect and PANSS Positive *r*(314) = .09, *P* = .13, (CIs −.03 .20)) was associated with positive symptoms.

Hypothesis 5 was not supported as negative symptoms were not associated with neglect (CTQ Neglect and PANSS Negative *r*(314) = .06, *P* = .31 (CIs −.06 .17)), or abuse (CTQ Abuse and PANSS Negative *r*(292) = .04, *P* = .52 (CIs −.08 .15)).

Hypothesis 6 predicted that abuse would be associated with hallucinations and with delusional beliefs, which was partially supported (CTQ Abuse and PSYRATS Hallucinations *r*(270) = .16, *P* = .008 (CIs .04 .28)); (CTQ Abuse and PSYRATS Delusions *r*(284) = .10, *P* = .085 (CIs −.02 .22)). The type of abuse (CPA, CEA, CSA) did not matter and all were associated with Hallucinations (CPA and PSYRATS Hallucinations *r*(286) = .15, *P* = .009 (CIs .04 .27), CEA and PSYRATS Hallucinations *r*(284) = .16, *P* = .008 (CIs .04 .27), CSA and PSYRATS Hallucinations *r*(282) = .16, *P* = .007 (CIs .04 .28)). Given the differences in reported rates of adversity between males and females the analyses were run again by sex and are reported in Supplementary file 1. These were not subject to formal analysis given the risk of type II error owing to the increased number of comparisons, and smaller sample sizes, however, it is evident that the relationship between CTQ total and PANSS total was greater in females (*r* *=* .4) than males (*r* *=* .14) and for females there was a greater association between CTQ with negative symptoms (*r* *=* .25 vs *r* *=* −.0.4), indicating that sex does affect the pattern of relationships between trauma and psychosis, to a degree.

As shown in table 5 for hypothesis seven, exposure to adversity was associated with negative views of self (Negative-Self and CTQ Total: *r*(277) = .27, *P* < .001 (CIs .16 .38)) and to a greater degree with abuse (Abuse Total: *r*(285) = .30, *P* < .001 (CIs .18 .40)) than neglect (Neglect Total: *r*(294) = .177, *P* = .002 (CIs .06 .29)). This was also the case with negative views of others (Negative-Other and CTQ Total: *r*(273) = .26, *P* < .001 (CIs .14 .37)) and with abuse (Negative-Other and Abuse Total: *r*(282) = .27, *P* < .001 (CIs .15 .37)) to a greater extent than with neglect (Negative-Other and Neglect Total: *r*(290) = .19, *P* = .001 (CIs .07 .30)).

**Table 5.** Descriptive Statistics (Mean and Standard Deviation) and Correlations Between Brief Core Schema Scale (BCSS; Negative-Self and Negative-Other Indices) and Childhood Trauma Questionnaire (CTQ)

Measures	*M* (SD)	1	2	3	4	5
		*r*, *P*, *N*	*r*, *P*, *N*	*r*, *P*, *N*	*r*, *P*, *N*	*r*, *P*, *N*
1. BCSS Negative-Self	7.03 (6.00)	1				
		439				
2. BCSS Negative-Other	8.84 (6.38)	.47**	1			
			.000			
		424	433			
3. CTQ Abuse	25.96 (13.26)	.30**	.27**	1		
			.000	.000		
		285	282	301		
4. CTQ Neglect	20.41 (8.69)	.18**	.19**	.64**	1	
			.002	.001	.000	
		294	290	292	314	
5. CTQ Total	46.34 (20.19)	.27**	.26**	.91**	.89**	1
			.000	.000	.000	.000
		277	273	292	292	292

Note: **Indicates *P* < .001.

*Indicates *P* < .01.

### Mediation Analysis

Mediation analysis investigated the indirect impact of negative beliefs about self and others on the relationship between Childhood Trauma (CT, abuse, and neglect) and psychosis symptoms. Mediation analysis was conducted based on 5000 bootstrapped samples using bias corrected and accelerated 95% CIs. Also, given the specific association between abuse and auditory hallucinations a second mediation analysis was undertaken to examine if the direct relationship was mediated by negative beliefs about self or others.

CT had a significant, direct path to psychotic symptomatology (*b* = .12, SE = .04, *P* = .005) and negative beliefs about self (*b* = .10, SE = .02, *P* < .000) and others (*b* = .09, SE = .02, *P* < .000). Negative beliefs about self, and others had a significant direct path to psychotic symptoms (*b* = .33, SE = .17, *P* = .049; *b* = .31, SE = .15, *P* = .041, respectively). The total model was significant (*R*^2^ = .07, *F*(1,268) = 19.65, *P* < .000). The total effect of CT on psychotic symptoms was significant (*b* = .18, SE = .04, *P* = .000) as was the direct effect (*b* = .12, SE = .04, *P* = .005). The indirect effect was significant (*b* = .06 Boot LLCI .02 Boot ULCI .10). Thus, negative-self and other beliefs mediated the relationship between CT and psychosis symptoms.

In a second analysis, abuse also had a significant direct path to negative-self-beliefs (*b* = .15, SE = .03, *P* < .000) and negative-other beliefs (*b* = .14, SE = .03, *P* < .000). The direct path from abuse to hallucinations was not significant (*b* = .13, SE = .07, *P* = .06), nor were the paths from negative-self (*b* = .22, SE = .17, *P* = .21) or negative-other beliefs to hallucinations (*b* = −.07, SE =.16, *P* = .67). The total model was significant (*R*^2^ = .02, *F*(1,247) = 6.2, *P* = .01). The total effect of abuse on hallucinations symptoms was significant (*b* = .16, SE = .06, *P* = .013) but not the direct effect (*b* = .13, SE = .07, *P* = .056). The indirect effect was not significant (*b* = .03 Boot LLCI −.036 Boot ULCI .08). Thus, negative-self and other beliefs did not mediate the relationship between abuse and hallucinations, which itself was not a significant relationship. Whilst abuse led to more negative views of self or others these beliefs did not contribute to the experience of hallucinations.

## Discussion

The relationship between adversity and symptoms of psychosis was examined in a large sample of people with TRP. Levels of abuse and neglect were comparable to those reported by other groups of people with psychosis indicating the contribution such experiences may play in people with TRP. There were high levels of reported neglect and abuse especially for females who were more likely to be abused but not neglected than males. Females reported especially high levels of severe sexual and emotional abuse.

Our results showed adversity was associated with higher levels of psychotic symptoms generally and more so with positive than negative symptoms. Moreover, abuse rather than neglect was associated with positive but not with negative symptoms. Abuse rather than neglect was associated with hallucinations but not delusions. Abuse and neglect were related to negative beliefs about the self and negative beliefs about others.

Most of the relationships were modest but the findings largely support previous work indicating that adversity contributes to people with psychosis experiencing distressing symptoms, especially, hallucinations. The mediation demonstrated a general relationship with CA and neglect, negative-self, and other views and overall psychotic symptoms^40^ but not in relation to the specific experience of abuse and hallucinations.

A number of limitations are evident. There is a lack of detail of the nature, duration, frequency, and perceived impact of the trauma events. The ages at which people experienced trauma which may have been important as the timing of stress has different effects on neurodevelopment, with early stress having a more severe and specific effect, creating a sensitization to later stressors in adolescence and adulthood. In addition, there is no account of later adult trauma and no assessment of the presence of current Post-Traumatic Stress Disorder, which is associated with lower quality of life, poorer functioning, higher levels of positive symptoms, general psychopathology, and poorer neurocognitive functioning.^55^

Whilst CBT for psychosis may address negative beliefs about self and others, the lack of mediation via negative belief about self and others indicates that these are not the key variables helping to contribute to hallucinations. It may be that other factors such as dissociation^11,56–59^ or impact on attachment relationships may be better candidates.

Despite these and other limitations, our work further demonstrates the important role of adversity in childhood to the later emergence of distressing and impacting symptoms of psychosis. This reminds us of the need to purposefully but sensitively ask about these issues. Trauma-informed care involves taking a good trauma history and appreciating the impact of trauma on a person’s presentation, including on psychotic symptoms. Given the high rate of reported abuse and neglect it would seem important that the consequences of trauma should be screened for, including post-traumatic stress disorder, complex PTSD, dissociative disorders, as well as the impact of depression and anxiety. This then provides a stronger rationale to provide treatment that reduces the impact of trauma with the plausible rationale that this can also reduce the frequency and distress of symptoms of psychosis. This has led to the suggestion that such individuals may be better suited to treatment with trauma focused therapies, like Eye Movement Desensitization and Reprocessing (EMDR)^60^ and trauma focused cognitive behavioral therapy (CBTtr).^19,61^ The use of trauma focused therapies^56,59,62^ has previously not been available to people with psychosis often for fear of worsening their psychotic symptoms.^63^

## Supplementary Material

sgad030_suppl_Supplementary_Tables_S1-S2
