# Supplementary material for: Childhood Trauma in Clozapine-Resistant Schizophrenia: Prevalence, and Relationship With Symptoms
Source: Schizophr Bull Open. 2023 Nov 13;4(1):sgad030. doi: 10.1093/schizbullopen/sgad030 (PMC11207680; doi:10.1093/schizbullopen/sgad030)
Supplement: sgad030_suppl_Supplementary_Tables_S1-S2 [file sgad030_suppl_Supplementary_Tables_S1-S2.docx]

Supplementary Table 1.

*Descriptive statistics (means, standard deviations) and correlations between Childhood Trauma Questionnaire (CTQ) and PANSS/PSYRATS symptoms of psychosis for female participants.*

| **Measures** |  | **1** | **2** | **3** | **4** | **5** | **6** | **7** | **8** |
| --- | --- | --- | --- | --- | --- | --- | --- | --- | --- |
|  | ***M (SD)*** | **r, p, N** | **r, p, N** | **r, p, N** | **r, p, N** | **r, p, N** | **r, p, N** | **r, p, N** | **r, p, N** |
| 1. Total CTQ | 52.72 | 1 |  |  |  |  |  |  |  |
|  | (24.24) | . |  |  |  |  |  |  |  |
|  |  | 82 |  |  |  |  |  |  |  |
| 2. CTQ Abuse | 31.29 | .95** | 1 |  |  |  |  |  |  |
|  | (16.47) | .000 | . |  |  |  |  |  |  |
|  |  | 82 | 86 |  |  |  |  |  |  |
| 3. CTQ Neglect | 21.46 | .91** | .76** | 1 |  |  |  |  |  |
|  | (9.20) | .000 | .000 | . |  |  |  |  |  |
|  |  | 82 | 82 | 84 |  |  |  |  |  |
| 4. PANSS Total | 82.27 | .40** | .40** | .35** | 1 |  |  |  |  |
|  | (13.50) | .000 | .000 | .001 | . |  |  |  |  |
|  |  | 82 | 86 | 84 | 89 |  |  |  |  |
| 5. PANSS Positive | 24.99 | .13 | .19 | .06 | .63** | 1 |  |  |  |
|  | (5.52) | .240 | .080 | .582 | .000 | . |  |  |  |
|  |  | 82 | 86 | 84 | 89 | 89 |  |  |  |
| 6. PANSS Negative | 18.25 | .25 | .28* | .20 | .60** | .27 | 1 |  |  |
|  | (5.61) | .024 | .010 | .064 | .000 | .012 | . |  |  |
|  |  | 82 | 86 | 84 | 89 | 89 | 89 |  |  |
| 7. PSYRATS Hallucinations | 24.26 | .011 | .07 | -.08 | .17 | .17 | .13 | 1 |  |
|  | (13.91) | .922 | .567 | .505 | .133 | .127 | .234 | . |  |
|  |  | 76 | 80 | 78 | 82 | 82 | 82 | 82 |  |
| 8. PSYRATS Delusions | 15.08 | -0.01 | -.02 | .04 | .38** | .36** | .07 | .133 | 1 |
|  | (5.61) | .943 | .828 | .706 | .000 | .000 | .518 | .242 | . |
|  |  | 78 | 82 | 80 | 85 | 85 | 85 | 79 | 85 |

*** indicates p <.001, * indicates p <.01*

*Suppl Table 2. Descriptive statistics (means, standard deviations) and correlations between Childhood Trauma Questionnaire (CTQ) and PANSS/PSYRATS symptoms of psychosis for male participants.*

| **Measures** |  | **1** | **2** | **3** | **4** | **5** | **6** | **7** | **8** |
| --- | --- | --- | --- | --- | --- | --- | --- | --- | --- |
|  | ***M (SD)*** | **r, p, N** | **r, p, N** | **r, p, N** | **r, p, N** | **r, p, N** | **r, p, N** | **r, p, N** | **r, p, N** |
| 1. Total CTQ | 43.86 | 1 |  |  |  |  |  |  |  |
|  | (17.83) | . |  |  |  |  |  |  |  |
|  |  | 210 |  |  |  |  |  |  |  |
| 2. CTQ Abuse | 23.82 | .88** | 1 |  |  |  |  |  |  |
|  | (11.09) | .000 | . |  |  |  |  |  |  |
|  |  | 210 | 215 |  |  |  |  |  |  |
| 3. CTQ Neglect | 20.03 | .88** | .59** | 1 |  |  |  |  |  |
|  | (8.57) | .000 | .000 | . |  |  |  |  |  |
|  |  | 210 | 210 | 221 |  |  |  |  |  |
| 4. PANSS Total | 82.50 | .14 | .18* | .07 | 1 |  |  |  |  |
|  | (14.08) | .043 | .007 | 314 | . |  |  |  |  |
|  |  | 210 | 215 | 221 | 227 |  |  |  |  |
| 5. PANSS Positive | 24.89 | .20* | .25** | .10 | .61** | 1 |  |  |  |
|  | (6.05) | .005 | .000 | .144 | .000 | . |  |  |  |
|  |  | 210 | 215 | 221 | 227 | 227 |  |  |  |
| 6. PANSS Negative | 19.53 | -.02 | -.04 | -.00 | .60** | .17* | 1 |  |  |
|  | (5.86) | .747 | .568 | .992 | .000 | .010 | . |  |  |
|  |  | 210 | 215 | 221 | 227 | 227 | 227 |  |  |
| 7. PSYRATS Hallucinations | 22.27 | .17 | .19* | .08 | .17 | .21* | -.01 | 1 |  |
|  | (13.41) | .022 | .007 | .249 | .017 | .003 | .862 | . |  |
|  |  | 185 | 190 | 194 | 200 | 200 | 200 | 200 |  |
| 8. PSYRATS Delusions | 14.27 | .09 | .14 | .01 | .41** | .40** | .14 | .33** | 1 |
|  | (5.24) | .209 | .045 | .893 | .000 | .000 | .037 | .000 | . |
|  |  | 197 | 202 | 207 | 212 | 212 | 212 | 188 | 212 |

*** indicates p <.001, * indicates p <.01*
